# Flexural Strength of Vitreous Ceramics Based on Lithium Disilicate and Lithium Silicate Reinforced with Zirconia for CAD/CAM

**DOI:** 10.1155/2022/5896511

**Published:** 2022-02-02

**Authors:** Hazel P. R. Corado, Pedro H. P. M. da Silveira, Vagner L. Ortega, Guilherme G. Ramos, Carlos N. Elias

**Affiliations:** ^1^Department of Materials Science, Instituto Militar de Engenharia-IME, Rio de Janeiro 22290-270, Brazil; ^2^São Leopoldo Mandic University, Campinas 13045-755, Brazil

## Abstract

The dental prosthesis market is rapidly evolving to meet patient and clinical demands. These new materials must have good flexural strength, toughness, aesthetic properties, and reliability in performance for structural applications. The present work aimed to compare the bending strength of 4 types of chairside lithium disilicate (Li_2_Si_2_O_5_) glass-ceramics used for dental prosthesis and to analyze the influence of heat treatment on the transformation of lithium metasilicate (Li_2_SiO_3_) into lithium disilicate. The three-ball test for the biaxial flexion test (B3B) was used. Weibull statistical analysis was used, and it showed that samples with a higher percentage of zirconia have a greater tendency to fail. The flexion tests showed that the addition of more than 10% of zirconia reduced the flexural strength. The heat treatment process improves and provides greater mechanical strength. The XRD results indicated that the samples with the lowest percentage of zirconia exhibited greater crystallinity and corroborated the microstructural analysis. SEM analyses showed a greater amount and elongated crystals of lithium disilicate when comparing samples with a higher percentage of zirconia. Therefore, samples with lower zirconia showed greater flexural strength than samples with higher additions of zirconia.

## 1. Introduction

The search for aesthetic and structural dentistry restorative treatment has been increasingly challenging, owing to the growing number of materials and techniques available for prosthetic rehabilitation.

Currently, the manufacture of dental prostheses has been carried out with CAD-CAM (computer-aided design and computer-aided manufacture) systems, which present excellent results and ease of execution. Using this technology, the technician can design and manufacture customized aesthetic abutments and all-ceramic or composite resin crowns since the molding stage is optional. CAD/CAM tools have allowed dentists to simplify laboratory steps and shorten their duration from a few weeks to just 1 day [1].

The CAD/CAM technology has led to the development of a wide range of ceramic materials for monolithic dental restorations, and producing presintered blocks with minimal defects and milling failures allows dental restorations to combine strength and aesthetics [2, 3]. Dental ceramics (zirconia and alumina) have excellent aesthetic, biocompatible, and resistance properties. However, the hardness and brittleness of the material result in crack formation during the loading and wear of the opposing teeth [4].

Researchers have tried to develop an ideal ceramic material having physical characteristics that are in harmony with the needs of the stomatognathic system and aesthetic properties. The translucency of glass-ceramics allows light to reflect very close to the dental structures, which is visually appealing and results in highly aesthetic restorations. Among the various materials developed for the manufacture of dental prostheses with CAD-CAM systems, lithium disilicate and lithium silicate glass-ceramics are worth mentioning. Lithium disilicate glass-ceramic (Li_2_Si_2_O_5_) is currently used for single and multiunit dental restorations. This ceramic is favored in cases requiring full crowns, ceramic laminates, and fragments because of its high substrate adhesion and flexural strength among available glass systems [5, 6]. The main advantage is its color being similar to natural teeth. Previous studies have shown the flexural strength for lithium-based glass-ceramics ranging from 300 to 520 MPa (8–11) and prosthesis survival rates ranging from 96% to 100% in 3 years [7, 8].

However, Li_2_Si_2_O_5_ lacks chemical stability in the oral cavity and presents mechanical properties degradation. The addition of oxide was carried out to improve its chemical and mechanical stability. Several oxides (Al_2_O_3_, K_2_O, ZnO, ZrO_2_, CaO, and P_2_O_5_) were tested to improve the lithium disilicate glass-ceramic properties for use as a restorative material in dentistry [9].

This study is important because the rapid flow of new dental materials on the market ensures that there is always a wide variety of options available to dentists and patients. This study aims to isolate and identify the glass-ceramic materials of four different brands in important ones less than 5% and greater than 10% of the zirconia content, with flexural strength as the main comparison parameter so that dentists can be more informed about which restorative materials to use in clinical practice.

## 2. Materials and Methods

### 2.1. Materials

The objective of the present work is to compare the bending strength and Vickers microhardness of 4 types of chairside CAD-CAM lithium silicate glass-ceramics used for dental prosthesis and analyze the influence of heat treatment on one of them. Celtra® Duo (Dentsply Sirona, USA), IPS e.max CAD (Ivoclar Vivadent Inc., Schaan, Liechtenstein), Suprinity (Vita Zahnfabrik, DE), and Rosetta SM (HASS, Korea) are commercially available as blocks for prosthetic dental manufacturing.

According to the blocks' manufacturers, the Celtra® Duo is available as a lithium metasilicate compound reinforced with zirconia. Celtra® Duo is mainly composed of 58% silica, lithium metasilicate, disilicate, and phosphate crystals, and 10% zirconia crystals in addition to other minor oxides and ingredients. The IPS e.max CAD is a lithium disilicate glass-ceramic, is more translucent than zirconia, and is composed of quartz, lithium dioxide, phosphor oxide, alumina, potassium oxide, and other components. The Vita Suprinity ceramic is a lithium disilicate glass-reinforced ceramic and has SiO_2_ (56–64%), Li_2_O (15–21%), ZrO_2_ (8–12%), and other minor oxides in lower percentages. The Rosetta SM ceramic is a lithium disilicate glass-reinforced ceramic for CAD-CAM systems.

Celtra Duo®, IPS e.max CAD®, Vita Suprinity®, and Rosetta SM® blocks are machined in a partially crystallized form. After machining, the crows received heat treatment for total crystallization and stress relief. Celtra Duo® has the double possibility of, after being machined, being polished and cemented on the patient's dental element; for this reason, group 2 did not undergo the crystallization process.

The samples were divided into 5 groups (Table 1). Ten samples of each group were machined. Samples from group 1 and group 2 are machined from Celtra Duo® blocks. The difference between group 1 and group 2 was that only samples from group 1 were heat-treated for the transformation of lithium metasilicate to lithium disilicate, full crystallization, and stress relief. The objective of Groups 1 and 2 was to analyze the influence of heat treatment, lithium metasilicate, lithium disilicate phase, and partially and full-crystallized silicate for mechanical properties.

The samples (*n* = 10/material) were classified into 5 groups according to Table 1: group 1, Celtra Duo® crystallized (Dentsply); group 2, Celtra Duo noncrystallized; Celtra Duo material is sold commercially with dual use. It is machined, then polished, and cemented into dental elements or the second option is, after machining, additional heat treatment can be carried out. Soon, it will be tested by the two options of use; group 3, IPS e.max CAD (Ivoclar Vivadent); group 4, Vita Suprinity (Wilcos); and group 5, Rosetta SM (HASS). The ceramics of groups 1, 2, and 4 are lithium silicate reinforced with more than 10%wt zirconia. Ceramics in groups 3 and 5 are lithium disilicate with less than 5% zirconia.

According to the manufacturers, Celtra Duo is mainly composed of crystals of 58% silica, lithium metasilicate, disilicate, and phosphate, and 10% zirconia crystals in addition to other oxides and smaller ingredients. IPS e.max CAD is a lithium disilicate glass-ceramic, more translucent than zirconia, and composed of quartz, lithium dioxide, phosphorus oxide, alumina, potassium oxide, and other components. The Vita Suprinity ceramic is a glass-reinforced lithium disilicate ceramic for CAD-CAM systems, has ZrO_2_ (8–12 %wt), SiO_2_ (56–64 %wt), Li_2_O (15–21 %wt), and other oxides in lower percentages. Rosetta SM ceramic is a ceramic reinforced with lithium disilicate glass for CAD-CAM systems such as e.max CAD.

### 2.2. Sample Preparation

Rectangular samples were cut from CAD-CAM blocks using a water-cooled, low-speed diamond saw using the IsoMet 1000 metallographic cutter (Buehler, Lake Bluff, IL, USA). From the block, 10 samples measuring 12 mm × 12 mm × 1.2 mm were cut according to ISO 6872:2015. After the cut was made, the sample surface was analyzed with a stereoscopic magnifying glass EK3ST (Eikonal Equip. Optics and Analytical, SP, Brazil) to check for possible cracks and fractures. The samples from groups 1, 3, 4, and 5 were heated for crystallization in a Programat 300 furnace (Ivoclar Vivadent) according to the manufacturer's recommendations, as described in Table 2.

### 2.3. Scanning Electron Microscopy (SEM)

After bending testing, the microstructure of the fractured ceramics was analyzed by scanning electron microscopy (Quanta FEG 250, Thermo Fisher Scientific, Massachusetts, USA). The MEV is equipped with a field-emission electron gun, operating at 5 and 10 kV in low-vacuum mode, with 10000x magnification for all samples. The objective of the analysis was to identify the microstructure of the material and evaluate the morphology of the crystals. Samples were received and cut without additional heat treatment and after heat treatment. All samples were treated with 10% hydrofluoric acid for 15 seconds, then coated with a thin layer of gold using a sputter (ACE600, Leica, Germany) for 30 min.

### 2.4. X-Ray Diffractometry (XRD)

XRD was used to determine the crystalline phases present before and after crystallization heat treatment. The measurements were performed using a high-resolution diffractometer (X'Pert MRD, Malvern Panalytical, Germany) equipped with a Co-k*α* X-ray tube, operating over the 2*θ* range 20–50° with a Ge primary monochromator. The XRD patterns collected were first indexed with the help of the PDF2 database to determine the crystalline phase(s) present on the diffractogram.

### 2.5. Compression Flexural Strength Test

Three ball test geometry for bending testing (B3B) was used. The possibility of using samples in the form of discs for the flexural testing with three spheres facilitates the preparation of the samples using CAD-CAM systems and the execution of the test. Both B3B test geometries (rectangular plates and cylindrical discs) were equally acceptable for testing and present similar flexural strength values for the same support radius [10, 11]. The B3B flexural test was performed using an EMIC DL200 machine (EMIC Co, PR, Brazil) with a speed test of 0.1 mm/s.

### 2.6. Vickers Microhardness (HV)

The specimens were prepared using the manual sanding method, using sandpapers of 600, 800, 1200, 2000, and 2500 mesh, to obtain a mirror surface and then, the test was carried out according to the ASTM C1327-15 standard. Ten impressions were made on the material by a pyramidal diamond indenter under a load of 1 kg for 15 s. The equipment used was a durometer (Shimadzu HMV-G series, Kyoto, Japan), used in conjunction with the AVPAK software. The hardness was calculated using the standard equation [12]:

(1) $$\begin{array}{c} \text{HV}=\frac{1.8544\cdot P}{d^{2}}, \end{array}$$

where *P* is the applied load, *d* is the diagonal length of the indenter impression, and 1.8544 is a constant geometrical factor for the diamond pyramid.

### 2.7. Statistical Analysis

Statistical analysis was performed using the Origin Pro 2021 and GraphPad Prism 9.1 software, adopting a significance level of 5%. Some studies report the statistical theory for brittle materials, where larger samples are more likely to fracture, as they have large defect strength limiters, producing lower strength values than small samples tested under the same loading conditions [13, 14]. The data obtained were compared using the Weibull test.

## 3. Results and Discussion

### 3.1. Microstructure (SEM)

**Figure 1:**
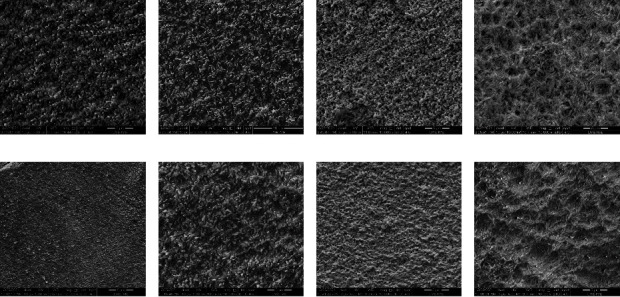
SEM micrographs of samples: (a) G1, Celtra Duo crystallized; (b) G2, Celtra Duo noncrystallized; (c) G3, e.max CAD noncrystallized; (d) G3, e.max CAD crystallized; (e) G4, Vita Suprinity noncrystallized; (f) G4, Vita Suprinity crystallized; (g) G5, Rosetta SM noncrystallized; (h) G5, Rosetta SM crystallized.

The physical and mechanical properties of glassy ceramics depend on several factors such as sintering processing, chemical composition, additives, thermal history, crystallinity, the composition of phases, phases percentages, and microstructure. The materials with crystallization after machinability and lower zirconia content, e-Max CAD, and Rosetta, G3 (Figure 1(d)) and G5 (Figure 1(h)) respectively, revealed that the microstructures of lithium disilicates were elongated crystals.

### 3.2. XRD Results

**Figure 2:**
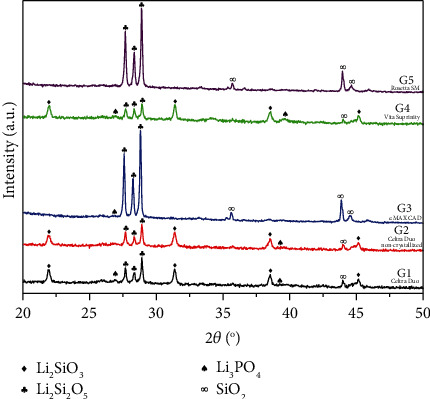
X-ray diffraction patterns of glass-ceramics from different manufacturers.

Figure 2 shows the XRD patterns of the different samples with 10% more zirconia and with the addition of up to 5% zirconia. The addition of zirconia to the ceramic Li_2_O-SiO_2_ influences the crystallization, preventing the growth of the grains, since ZrO_2_ influences the crystallization kinetics of the crystalline phases. Ceramics with a high zirconia content G1, G2, and G4 (Figures 1(a), 1(b), and 1(f)) reveal an interconnected microstructure of small plate-like crystals. Materials with crystallization after machinability and lower zirconia content, e.max CAD and Rosetta SM, G3 (Figure 1(d)) and G5 (Figure 1(h)), respectively, revealed that the microstructures of lithium disilicates were elongated crystals.

### 3.3. Flexural Bending Strength

**Figure 3:**
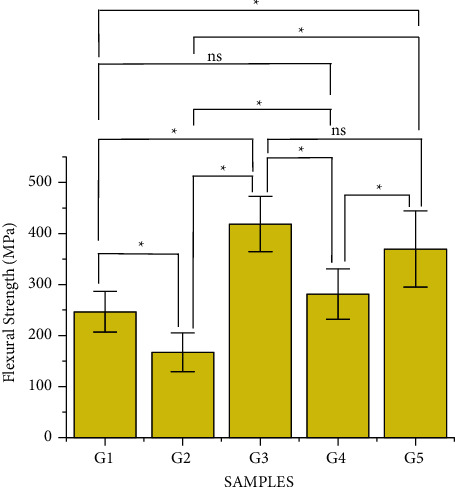
Bar graph of the flexural strength of ceramics. Analysis of variance with Tukey's multiple comparison test was used to compare the flexural strength of the ceramics in this study. The (^*∗*^) represents the significant difference between the groups, and (ns) represents the nonsignificant values.

Table 3 and Figure 3 show that the e.max CAD (G3) had the highest flexural strength (418.22 ± 51.21 MPa) followed by Rosetta SM (369.59 ± 71.02 MPa). The noncrystallized Celtra Duo (G2) had the lowest flexural strength (167.18 ± 35.88 MPa).

**Figure 4:**
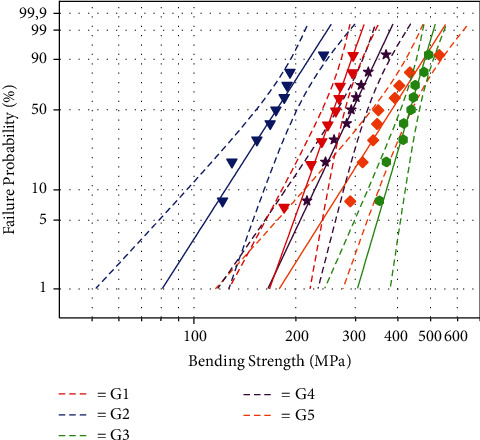
Weibull probability plot of the flexural strength of lithium disilicate ceramics.

Figure 4 and Table 3 show the results of the Weibull analysis for the flexural strength results (95% CI). The samples from the IPS e.max group (G3) had the highest Weibull modulus (*m* = 11.86), and the noncrystallized Celtra Duo group (G2) had the lowest value (*m* = 5.46).

### 3.4. Vickers Microhardness Results

**Figure 5:**
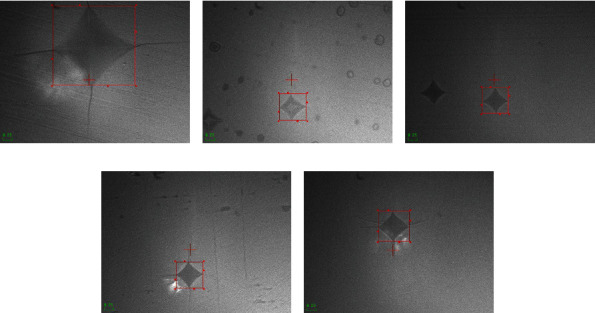
Vickers impression on lithium disilicate ceramics: (a) G1, Celtra Duo crystallized; (b) G2, Celtra Duo noncrystallized; (c) G3, e.max CAD crystallized; (d) G4, Vita Suprinity; (e) G4, Rosetta SM crystallized.

Figure 5 shows the marks obtained in the Vickers hardness test for each sample.

**Figure 6:**
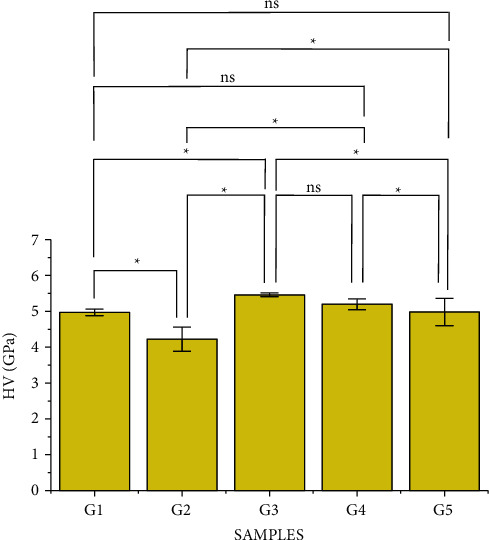
Bar graph of Vickers hardness of ceramics. Analysis of variance with Tukey's multiple comparison test was used to compare the hardness of ceramics in this study. ^∗^ represents the significant difference between the groups and (ns) represents the nonsignificant values.

Figure 6 shows Tukey's multiple comparison analysis, where it was verified that there was no significant difference between groups G1 and G4, G1 and G5, and G3 and G4. Group G2, noncrystallized, is the group with the lowest hardness.

**Figure 7:**
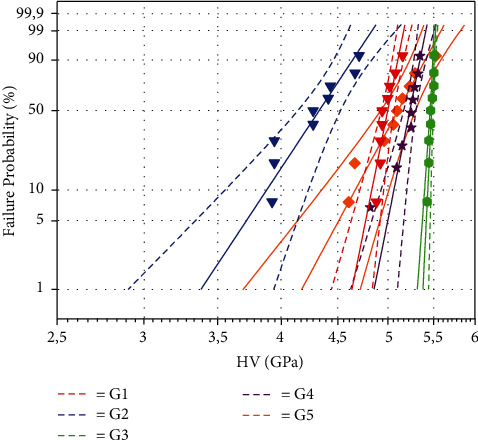
Weibull probability plot of the Vickers hardness of lithium disilicate ceramics.

The Weibull modulus shows the scatter results. As the Weibull modulus increases, the more homogeneous is the hardness of glass ceramics, and consequently, the more predictable the behavior of the material becomes.  Figure 7 and Table 4 show the results of the Weibull analysis (95% CI) for Vickers microhardness. The samples from the IPS e.max group (G3) had the highest Weibull modulus (*m* = 231.53) and the highest Vickers microhardness value of 5.46 GPa, and the lowest value was found for the noncrystallized Celtra Duo group (G2), with HV = 4.22 GPa and *m* = 17.10.

## 4. Discussion

The flexural strength values obtained in the present work were close to those found in the literature, which are 416.1 ± 50.1 MPa and 365.1 ± 46.0 MPa. The difference was not significant between both groups. Literature results showed that there was no statistically significant difference in flexural strength between IPS e.max CAD and Rosetta SM. The literature studies and the present work used the same size, shape specimens, and standard recommendations [15].

The results showed that the Li_2_O-SiO_2_ ceramics heat treatment influenced the flexural strength. Although in the present work, the heat treatment of samples of group 1 was only 90 seconds at 820°C, the flexural strength of Celtra Duo after heat treatment (G1) was higher than that of the same glass-ceramic without heat treatment (167 MPa) (G2). Described that the crystalline fraction can vary according to the temperature and the time of nucleation and crystal growth, it is known that the thermal treatments allow to obtain the desired microstructure and to optimize the glass-ceramic properties, increasing the mechanical resistance of brittle materials [16]. Details of the effect of thermal treatment parameters on the transformation of lithium metasilicate (Li_2_SiO_3_) into lithium disilicate (Li_2_Si_2_O_5_) and the resulting mechanical properties have been shown in the literature [16].

The ceramic cooling from high temperature to room temperature is a drastic treatment that all too often leads to distortion and even crack nucleation. Consequently, the mechanical properties of brittle materials depend on residual stresses developed from thermal stress and phase transformation. To reduce thermal stresses, the ceramic shall be cooling at a low rate.

The materials with crystallization after machinability and lower zirconia content, e.max CAD and Rosetta, G3 (Figure 1(d)) and G5 (Figure 1(h)), respectively, revealed that the microstructures of lithium disilicates were elongated crystals. Li_2_O-SiO_2_ ceramics with less than 5% ZrO_2_ content present 70% of the crystalline phase in the vitreous ceramic. However, glass-ceramics with more than 10% reinforcement of ZrO_2_ have a lower percentage of crystalline phase (40–50% of the crystalline phase) [17–19].

Material manufacturers reported that the zirconia crystals addition increases the strength of Li_2_O-SiO_2_ ceramics. A previous study [20] showed the opposite, stating that there are no clinical advantages for Li_2_O-SiO_2_ reinforced with ZrO_2_. Another study [10] compared the mechanical properties of e.max CAD with Celtra Duo. The e.max CAD showed significantly higher biaxial strength and fracture toughness than Celtra Duo, corroborating the results of bending strength obtained experimentally in this work.

Dentists should carefully choose dental ceramics for use in clinical practice. It is important to analyze the flexural strength, translucency parameters, color, fracture toughness, elasticity module, and biocompatibility, among other factors. The optimization of these factors in ceramics will properly promote their use, making it possible to provide satisfactory patient treatment [11, 21].

The Vickers microhardness values are in agreement with those found in the literature, 5.35 GPa (545.68 HV) for the G3 group [22] as well as G1 (5.3) and G2 (4.54), showing that the material without the additional crystallization has lower hardness [23]. It was observed that ceramics with the highest percentage of crystalline phase presented secondary radial cracks and lateral cracks in a greater quantity. When analyzing the glass-ceramics with a low crystalline fraction, few secondary radial cracks were observed; however, they were larger and more defined primary cracks [24].

## 5. Conclusions

Based on the results obtained and within the limitations of this study, the following conclusions were made. Lithium disilicate glass-ceramics (e.max CAD and Rosetta SM) showed greater flexural strength than lithium silicate ceramics with zirconia additions (Celtra Duo crystallized or noncrystallized and Vita Suprinity); glass ceramics e.max CAD has the highest Weibull modulus, which means this ceramic has less dispersion and homogeneity of flexural strength, hardness, and a greater chance of predicting failure. A clinical evaluation is necessary for an adequate indication of the material, and the addition of zirconia decreased the crystallinity of the ceramic, increasing the number of microcracks on the surface and decreasing the flexural strength and its hardness is a little lower than the material with a lower percentage of zirconia. However, the additional crystallization process provides greater mechanical strength. The clinical assessment of the patient and the needs of each case will guide which material is most suitable for each situation. Therefore, it is important to highlight the mechanical properties of each commercially available material to choose the most suitable one.

## Figures and Tables

**Table 1:** General description of the materials in this study, manufacturers, and composition.

Group	Brand	Manufacturer	Chemical composition	Lot #
G1 and G2^*∗*^	Celtra Duo	Dentsply	SiO_2_, Li_2_O, P_2_O_5_, ZrO_2_, Al_2_O_3_, K_2_O, CeO_2_, Na_2_O, Tb_4_O_7_, V_2_O_5_, Pr_6_O_11_, Cr, Cu, Fe, Mg, Mn, Si, Zn, Ti, Zr, and Al	16000093
G3	IPS e.max CAD	Ivoclar Vivadent	SiO_2_, Li_2_O, P_2_O_5_, ZrO_2_, Al_2_O_3_, K_2_O, ZnO, MgO, and color pigments	W35197
G4	Suprinity	Vita Zahnfabrik	SiO_2_, Li_2_O, P_2_O_5_, ZrO_2_, Al_2_O_3_, K_2_O, CeO_2_, and color pigments	51657
G5	Rosetta SM	HASS	Li_2_O, SiO_2_, P_2_O_5_, B_2_O_3,_ and other oxides	ACE24HG2101

**Table 2:** Parameters used in the crystallization of the samples.

	G1	G3	G4	G5
Predrying	2 min	—	—	—
Drying	2 min	—	—	—
Preheating	2 min	—	—	—
Starting temperature (°C)	500	403	400	400
Temperature increase	55°C/min	t1 90°C/min t2 30°C/min	55°C/min	60°C/min
Burning temperature	—	T1 820°C T2 840°C	T 840°C	T 840°C
Final temperature	820°C	—	—	700°C
Vacuum	Off	Vacuum1: 550°C; e 1022°C Vacuum2: 820°C; e 1508°C	Vacuum1: 410°C Vacuum2: 840°C	On: 550°C Off: 840°C
Waiting time	1.5 min	—	4 min	—
Closing time	—	6 min	—	—
Maintenance time	—	H1: 10 sec H2: 7 min	8 min	10 min
Cooling rate (°C/min)	3 min	—	—	—

**Table 3:** Flexural strength values and Weibull modulus for glass-ceramics.

Group	Manufacturer	Flexural strength (MPa)	Weibull modulus
G1	Celtra Duo crystallized	246.79 ± 39.81	9.74
G2	Celtra Duo noncrystallized	167.18 ± 37.82	5.46
G3	e.max CAD	418.22 ± 53.98	11.86
G4	Vita Suprinity	281.23 ± 49.43	7.37
G5	Rosetta SM	369.59 ± 74.86	5.54

**Table 4:** Vickers microhardness values and Weibull modulus for glass-ceramics.

Group	Manufacturer	HV (GPa)	Weibull modulus
G1	Celtra Duo	4.97 ± 0.09	56.46
G2	Celtra Duo (noncrystallized)	4.22 ± 0.34	17.10
G3	e.max CAD	5.46 ± 0.05	231.53
G4	Vita Suprinity	5.20 ± 0.15	56.48
G5	Rosetta SM	4.98 ± 0.38	20.98

## Data Availability

The data of SEM, XRD, Vickers hardness, and statistical analyses used to support the conclusions of this study are included in the article.
